# Analysis of protein secretion in *Bacillus subtilis* by combining a secretion stress biosensor strain with an in vivo split GFP assay

**DOI:** 10.1186/s12934-023-02199-8

**Published:** 2023-10-07

**Authors:** Patrick Lenz, Patrick J. Bakkes, Carolin Müller, Marzena Malek, Roland Freudl, Marco Oldiges, Thomas Drepper, Karl-Erich Jaeger, Andreas Knapp

**Affiliations:** 1 Institute of Molecular Enzyme Technology, Heinrich Heine University Düsseldorf, Forschungszentrum Jülich, 52425 Jülich, Germany; 2 Institute of Biotechnology, RWTH Aachen University, 52074 Aachen, Germany; 3 Institute of Bio- and Geoscience IBG-1: Biotechnology, Forschungszentrum Jülich, 52425 Jülich, Germany; 4 Castrol Germany GmbH, 41179 Mönchengladbach, Germany

**Keywords:** *Bacillus subtilis*, Protein secretion, Split GFP assay, Secretion stress biosensor, Online detection

## Abstract

**Background:**

*Bacillus subtilis* is one of the workhorses in industrial biotechnology and well known for its secretion potential. Efficient secretion of recombinant proteins still requires extensive optimization campaigns and screening with activity-based methods. However, not every protein can be detected by activity-based screening. We therefore developed a combined online monitoring system, consisting of an in vivo split GFP assay for activity-independent target detection and an mCherry-based secretion stress biosensor. The split GFP assay is based on the fusion of a target protein to the eleventh β-sheet of *sf*GFP, which can complement a truncated *sf*GFP that lacks this β-sheet named GFP1-10. The secretion stress biosensor makes use of the CssRS two component quality control system, which upregulates expression of *mCherry* in the *htrA* locus thereby allowing a fluorescence readout of secretion stress.

**Results:**

The biosensor strain *B. subtilis* PAL5 was successfully constructed by exchanging the protease encoding gene *htrA* with *mCherry via* CRISPR/Cas9. The *Fusarium solani pisi* cutinase Cut fused to the GFP11 tag (Cut11) was used as a model enzyme to determine the stress response upon secretion mediated by signal peptides SP_Pel_, SP_Epr_ and SP_Bsn_ obtained from naturally secreted proteins of *B. subtilis*. An in vivo split GFP assay was developed, where purified GFP1-10 is added to the culture broth. By combining both methods, an activity-independent high-throughput method was created, that allowed optimization of Cut11 secretion. Using the split GFP-based detection assay, we demonstrated a good correlation between the amount of secreted cutinase and the enzymatic activity. Additionally, we screened a signal peptide library and identified new signal peptide variants that led to improved secretion while maintaining low stress levels.

**Conclusion:**

Our results demonstrate that the combination of a split GFP-based detection assay for secreted proteins with a secretion stress biosensor strain enables both, online detection of extracellular target proteins and identification of bottlenecks during protein secretion in *B. subtilis*. In general, the system described here will also enable to monitor the secretion stress response provoked by using inducible promoters governing the expression of different enzymes.

**Supplementary Information:**

The online version contains supplementary material available at 10.1186/s12934-023-02199-8.

## Introduction

The Gram-positive soil bacterium *Bacillus subtilis* is one of the most important microorganisms for industrial recombinant protein production [1, 2] and thus became a major workhouse in biotechnology as outlined in a recent review [3]. The main reasons include the GRAS (generally recognized as safe) status of derived products, a non-biased codon usage, easy fermentation in large quantities and genetic accessibility through natural competence [2, 4–6]. *B. subtilis* harbors a potent Sec secretion machinery allowing for secretion yields of up to 25 g protein per liter culture medium [2, 7]. Downstream processing of secretory proteins is relatively simple and cost-effective because cell lysis and subsequent purification methods are not required [8]. Most proteins are secreted by *B. subtilis* in an unfolded conformation using the signal recognition particle (SRP)-dependent general secretion (Sec) pathway [9, 10]. Sec-secreted proteins possess N-terminal signal peptides with a tripartite domain structure consisting of a positively charged N-region followed by a hydrophobic H-region and a polar, uncharged C-region with the latter containing a signal peptidase I (SPase I) recognition site [11]. The SRP recognizes and binds the N-terminal signal peptide as it emerges from the ribosome tunnel. After interaction with the membrane-bound receptor FtsY, the ribosome-nascent chain complex is transferred to the SecYEG pore through which the target protein secretion is coupled to translation. Finally, a type I SPase cleaves the signal peptide prior to folding of the mature protein [10, 12].

At present, a combination of signal peptide and heterologous target protein resulting in an optimal secretion efficiency cannot be predicted. Thus, the most suitable signal peptide has to be identified experimentally. For this, libraries are used consisting of homologous or heterologous signal peptides [13, 14] or of mutants obtained from a specific signal peptide [15]. Such libraries are often analyzed by high-throughput screening using enzymatic activity assays with surrogate-substrates allowing for photometric or fluorometric detection as it is known e.g. for lipases [16] or proteases [17]. Such enzymatic activity assays are not available for all target proteins; we therefore adapted the split GFP technology for activity-independent in vitro detection of secreted and in vivo detection of cytoplasmic target proteins in *B. subtilis* [18, 19]. To this end, the relatively short eleventh β-sheet of GFP is C-terminally fused to a target protein (GFP11 tag). For detection, a truncated non-fluorescent *sf*GFP that lacks the eleventh β-sheet also called GFP1-10 is used. GFP1-10 and GFP11 tag can reconstitute a holo-GFP, whose fluorescence can be easily detected spectrophotometrically [20–22].

However, split GFP-based detection does not consider cellular stress caused by target protein secretion, which may exert adverse effects [23]. Therefore, transcription factor-dependent secretion stress biosensors were developed for industrially relevant organisms such as *Corynebacterium glutamicum* or *B. subtilis* [24, 25]. In *B. subtilis*, this biosensor is based on the CssRS two-component quality control system, which recognizes misfolded and aggregated proteins at the interface of the cell membrane and peptidoglycan and subsequently up-regulates the expression of protease genes *htrA* and *htrB* [23, 26, 27]. Genomic replacement of *htrA* with a fluorescence reporter gene enables the quantification of cellular secretion stress *via* a fluorescence readout [25].

In the present study, we established strain *B. subtilis* PAL5 which harbors a mCherry-based secretion stress biosensor. Additionally, a split GFP assay was developed allowing for the simultaneous and easy-to-perform determination of secreted target protein amount and the corresponding stress response in *B. subtilis* PAL5. The cutinase Cut from *Fusarium solani pisi* served as the model protein [28], which can be detected with a spectrophotometric activity assay using *p-*nitrophenyl palmitate as the substrate after secretion [29]. Both sensor systems were separately evaluated using a BioLector microbioreactor for online monitoring of cutinase secretion fused to signal peptides SP_Pel_, SP_Epr_, and SP_Bsn_ obtained from *B. subtilis* secretory proteins. The combined systems enabled the identification of cutinase variants by monitoring secretion. Finally, a library of 173 different *B. subtilis* signal peptides fused to the cutinase was screened for enzymatically active secreted protein and secretion stress response to evaluate a putative relation between both processes.

## Materials and methods

### Bacterial strains, plasmids, media and growth conditions

All experiments were performed with the protease-deficient secretion stress biosensor strain *B. subtilis* PAL5, which is based on the protease-deficient strain *B. subtilis* DB430 [30]. The plasmids used here are described in Table 1. All *B. subtilis* strains were cultivated at 30 °C in enriched LB medium (1% (w/v) NaCl, 8% (w/v) tryptone, 0.5% (w/v) yeast extract, 1% (w/v) glucose) containing 50 µg/ml kanamycin for maintenance of plasmid pBSMul1 [31] and derivatives. *E. coli* DH5α [32] was used for molecular cloning and *E. coli* BL21(DE3) [33] for GFP1-10 production. The bacteria were cultivated at 37 °C in LB medium (1% (w/v) NaCl, 1% (w/v) tryptone, 0.5% (w/v) yeast extract) containing 100 µg/ml ampicillin for maintenance of plasmid pET22b-sfGFP1-10 for GFP1-10 production or 50 µg/ml kanamycin for maintenance of plasmids based on the CRISPR/Cas9 plasmid pJOE8999.1 [34]. Transformation was carried out using naturally competent *B. subtilis* cells [35] and chemically competent *E. coli* cells [36].

**Table 1:** Plasmids used in this study

Name	Description	Source
pBSMul1	*E. coli-B. subtilis* shuttle vector, P_HpaII_, secretion signal (*SPlipA*), ColE1, *rep*^*B*^, *Km*^*r*^, *Amp*^*r*^	[31]
pET22b-*sfGFP1-10*	pET22b(+) containing the truncated *sfGFP1*-*10* under control of P_*T7*_	[18]
pBSMul1lipA_SPBox	Signal peptide (SP) library based on pBSMul3 signal peptide library from [13] containing HindIII–XbaI inserts of the lipase *lipA* from *B. subtilis* with all 173 predicted *B. subtilis* Sec signal peptides	[18]
pJOE8999.1	*E. coli-B. subtilis* shuttle vector, *pUCori, rep pE194ts, cas9, Km*^*R*^, *tracrRNA, lacPOZ*	[34]
pJOE-sgRNA:htrA	pJOE8999.1 with inserted sgRNA for *htrA* locus (5’-TACATCCGTGAGGTCGCTTC-3’) between BsaI restriction sites	This study
pJOE-sgRNA:htrA_H1H2	pJOE-sgRNA:htra with 3300 bp SfiI-SfiI fragment harboring the regions upstream and downstream of *htrA* required for homologous recombination and *htrA* (5’-*proG-htrA-ykcC*-3’).	This study
pJOE-sgRNA:htrA-htrA∷PhtrA-mCherry	pJOE-sgRNA:htrA-H1H2 with *mCherry* inserted between *proG-P*_*htrA*_ and *ykcC* using SLIC.	This study
pJT’Tmcs-mCherry	*Amp*^*R*^, *Gm*^*R*^, P_*tac*_, RBS, *mCherry*	[39]
pBS-4nt-SP_Pel_-Cut11	pBSMul1 containing an 827 bp NdeI–XbaI *SPpel*-*cut*-*11* fragment	[29]
pBS-4nt-SP_Epr_-Cut11	pBSMul1 containing an 845 bp NdeI–XbaI *SPepr*-*cut*-*11* fragment	[29]
pBS-4nt-SP_Bsn_-Cut11	pBSMul1 containing an 848 bp NdeI–XbaI *SPbsn*-*cut*-*11* fragment	[29]
pBS-Xnt-SP_Pel_-Cut11	pBS-4nt-SP_Pel_-Cut11 with a spacer extended by the insertion of 1–8 adenine(s) at the 5’-end from 4 to 12 nt	[29]
pBS-Xnt-SP_Epr_-Cut11	pBS-4nt-SP_Epr_-Cut11 with a spacer extended by the insertion of 1–8 adenine(s) at the 5’-end from 4 to 12 nt	[29]
pBS-Xnt-SP_Bsn_-Cut11	pBS-4nt-SP_Bsn_-Cut11 with a spacer extended by the insertion of 1–8 adenine(s) at the 5’-end from 4 to 12 nt	[29]
pBSMul1cut11_SPBox	Signal peptide (SP) library based on pBSMul3 signal peptide library from [18] containing HindIII–XbaI inserts of the cutinase *cut* from *F. solani pisi* with all 173 predicted *B. subtilis* Sec signal peptides and the GFP11 tag sequence at the 3’-end of *cut*	This study
pBSMul1_SPBox_SP_DacB_-Cut11	pBSMul1cut11_SPBox variant with signal sequence from DacB and 657 bp EcoRI-XbaI *cut-gfp11* fragment	This study
pBSMul1_SPBox_SP_Mpr_-Cut11	pBSMul1cut11_SPBox variant with signal sequence from Mpr and 657 bp EcoRI-XbaI *cut-gfp11* fragment	This study
pBSMul1_SPBox_SP_NamZ_-Cut11	pBSMul1cut11_SPBox variant with signal sequence from NamZ and 657 bp EcoRI-XbaI *cut-gfp11* fragment	This study
pBSMul1_SPBox_SP_NamZ*_-Cut11	pBSMul1cut11_SPBox variant with signal sequence from NamZ and 657 bp EcoRI-XbaI *cut-gfp11* fragment. Amino acid exchange A252S in GFP11-tag.	This study

### Recombinant DNA techniques

Standard DNA techniques were performed as described [36]. Kits for DNA purification were obtained from Analytic Jena (Jena, Germany), kit for isolation of genomic DNA from Qiagen (Hilden, Germany) and enzymes from Thermo Fisher Scientific (St. Leon-Roth, Germany).

### Construction of secretion stress biosensor strain *B. subtilis* PAL5

Strain construction was performed with the CRISPR/Cas9 plasmid pJOE8999.1 methodology as described previously [34].

#### Plasmid construction

For the targeted double-strand break in the *htrA* locus and the subsequent integration of the fluorescence reporter gene *via* homologous recombination, the CRISPy-web tool [37] was used for the identification of a suitable protospacer adjacent motif (5’-AGG-3’) and the corresponding spacer sequence (5’-TACATCCGTGAGGTCGCTTC-3’). For spacer sequence incorporation into plasmid pJOE8999.1, the oligonucleotides htrA-sgRNA1 (5’-AAACGAAGCGACCTCACGGATGTA-3’) and htrA-sgRNA2 (5’-TACGTACATCCGTGAGGTCGCTTC-3’), which were designed with 5’-end overhangs matching BsaI restriction sites, were hybridized by mixing in a molecular ratio of 1:1 and heating to 99 °C for 10 min prior to cooling down with 1 °C/min. Subsequently, the hybridized DNA was ligated into the BsaI hydrolyzed pJOE8999.1 resulting in plasmid pJOE-sgRNA:htrA. Successful integration was checked *via* blue-white screening [38] and subsequent sequencing with the primer pJOEInsertfw (5’- CTAAAGCTTAGGCCCAGTCGAAAG-3’). For homologous recombination after CRISPR/Cas9 induced double-strand break, the *htrA* locus and, additionally, the up- and downstream regions (each approx. 1 kb) were amplified as a SfiI fragment with primers fw-htrA-H1 (5’- TATAGGGTCGACGGCCAACGAGGCCGAATCCTCTTTCAAGGATTC-3’) and rev-htrA-H2 (5’- CTTAATCTAGAAAGGCCTTATTGGCCCTGATGAAAGGCTCGCCGGAG-3’) from isolated genomic *B. subtilis* DB430 DNA by PCR. The resulting DNA fragment was ligated into the SfiI hydrolyzed vector pJOE-sgRNA:htrA resulting in the homologous recombination cassette containing plasmid pJOE-sgRNA:htrA_H1H2. For replacement of *htrA* with the *mCherry* gene in the homologous recombination cassette, the plasmid pJOE-sgRNA:htrA_H1H2 was amplified *via* PCR without *htrA* thereby excluding *htrA* using primers fw-htrA-H2 (5’- GATGAGCTCTACAAATGACCTAGTGTAGGGACATAATGCCTCAGGCC-3’) and rev-SLIC-PhtrAfull (5’-CATGAATTCGATATCAAGCTTATCCATCATGTTCACTCC-3’). In addition, the *mCherry* gene from plasmid pJT’Tmcs-mCherry [39] was amplified with primers fw-SLIC-mCherry (5’- ATAAGCTTGATATCGAATTCATGGTGAGCAAGGGCGAG-3’) and rev-SLIC-mCherry (5’- GGTCATTTGTAGAGCTCATCTTACTTGTACAGCTCGTC-3’) before both DNA fragments were united to the plasmid pJOE-sgRNA:htrA-htrA∷PhtrA-mCherry by SLIC cloning [40].

#### Genomic integration of *mCherry* into *htrA* locus

*B. subtilis* DB430 was transformed with the newly constructed plasmid pJOE-sgRNA:htrA-htrA∷PhtrA-mCherry and was plated on LB agar plates containing 50 µg/ml kanamycin for plasmid maintenance and 0.2% (w/v) mannose for *cas9* induction. Thereafter, Cas9 produced a double-strand break in the *htrA* locus due to the previously cloned spacer sequence, which was repaired by homologous recombination between the genome and the plasmid-encoded homologous recombination cassette. In this process, the genomic *htrA* gene was replaced by the *mCherry* gene. After incubation at 37 °C for 16 h, clones were picked and transferred to fresh LB agar plates and incubated for 24 h at 50 °C, twice. Clones were checked for plasmid loss prior to amplification of the genomic segment of potential *mCherry* integrants *via* PCR with the primer pair fw-htrA-H1/rev-htrA-H2 and subsequent DNA sequencing.

### Construction of Cut11 signal peptide library

The plasmid pBS-4nt-SP_Pel_-Cut11 [29] was used to construct the Cut11 signal peptide library. The *cut11* gene was extracted by hydrolysis with EcoRI and XbaI and was ligated into the likewise hydrolyzed signal peptide library plasmid pBSMul1lipA_SPBox harboring a constitutive P_*HpaII*_ promoter [18]. After transformation, about 2000 single *E. coli* DH5α colonies were washed off from agar plates and plasmids were isolated to generate a library containing all 173 different signal peptides. This number of clones should result in a coverage of > 99% [41] of all signal sequences fused to *cut11*. *B. subtilis* PAL5 was transformed with this library and cultures grown from single colonies were analyzed regarding enzymatic activity towards *p*NPP, split GFP and mCherry fluorescence.

### Online cultivation and fluorescence measurements of *cut11* expression cultures in a biolector microbioreactor system

#### Expression cultures

For *cut11* expression cultures, 1 mL enriched LB medium was inoculated with a single *B. subtilis* transformant and grown overnight at 30° C and 1100 rpm in a FlowerPlate®. The overnight cultures were used to inoculate 1 mL expression medium in each FlowerPlate® well to an optical density (OD_580nm_) of 0.05. Cells were then cultivated in BioLector microbioreactor (m2p-labs, Baesweiler, Germany) at 30 °C and 1100 rpm for 24 h.

#### Online measurements

Cell growth was measured as light scattering signal at λ = 620 nm. For online monitoring of secretory Cut11 production, a 3% (v/v) GFP1-10 solution was added after 16 h of cultivation and the fluorescence accompanying the reconstitution of holo-GFP was measured using the eYFP filter (λ_Ex_ = 508 nm, λ_Em_ = 532 nm). GFP1-10 was produced in *E. coli* BL21(DE3) using pET22b-GFP1-10 in inclusion bodies, which were purified as described previously [18]. For obtaining GFP1-10 solution, the purified inclusion body fraction was dissolved in 9 M urea (1 ml urea per 75 mg of protein). Cellular stress caused by cutinase secretion was monitored using the mCherry filter (λ_Ex_ = 580 nm, λ_Em_ = 610 nm). All measurements were carried out at a time interval of 15 min.

### Cut11 signal peptide screening

#### Expression cultures

The Cut11 signal peptide screening was carried out in microtiter plates (MTPs). 150 µL enriched LB medium were inoculated with a single *B. subtilis* PAL5 library transformant and grown at 30 °C and 900 rpm. For expression cultures 150 µl enriched LB medium was inoculated with 5 µl of the overnight cultures prior to cultivation at 30 °C and 900 rpm for 24 h. For in vivo split GFP assay, 3% (v/v) GFP1-10 solution were added into culture broth after 16 h of cultivation.

#### Offline fluorescence measurements

For offline fluorescence measurements, the cultures were diluted 10-fold with 100 mM Tris-HCl pH 8 prior to the measurements in the Tecan Infinite M1000 Pro microplate reader (Tecan, Männedorf, Switzerland). The GFP fluorescence obtained by holo-GFP reconstitution was measured with the following parameters: λ_Ex_ = 485 nm (bandwidth 10 nm), λ_Em_ = 505–550 nm (5 nm steps, bandwidth 5 nm, gain 120). The emission maximum at 510 nm was used for analysis and was normalized to the cell density (OD_580_) for calculation of fluorescence units. Additionally, the secretion stress signal, represented by mCherry fluorescence, was read out with the following parameters: λ_Ex_ = 585 nm (bandwidth 10 nm), λ_Em_ = 605–650 nm (5 nm steps, bandwidth 5 nm, gain 150). The emission maximum at 610 nm was used for analysis and normalized to the cell density (OD_580_) for calculation of fluorescence units.

##### Determination of Cut11 activity

For determination of the enzymatic activity of Cut11, the chromogenic substrate *p*-nitrophenyl palmitate (*p*NPP, Sigma-Aldrich/Merck, Darmstadt, Germany) was used [42]. After cultivation, cultures were separated into supernatant and cell pellet by centrifugation. The supernatants were diluted 40-fold with 100 mM Tris-HCl pH8 and subsequently, 10 µl of the dilution were mixed with 190 µl substrate solution (47.22 mM K_2_HPO_4_, 2.77 mM KH_2_HPO_4_, 1.11 mg/ml gum arabic, 2.3 mg/ml sodium deoxycholic acid, 3 mg/ml *p-*nitrophenyl palmitate), incubated at 37 °C and the change of absorption at 410 nm was measured for 15 min using the SpectraMax 250 plate reader (Molecular Devices, Biberach an der Riss, Germany). The volumetric activity (U/ml) was calculated using the molar extinction coefficient of *p*NP (15,000 M^-1^ cm^-1^ for the used reaction conditions) and normalized to the cell density (OD_580_).

##### *In silico* analyses

Domain structures of SP_Pel_, SP_Epr_ and SP_Bsn_ were predicted with SignalP 6.0 [43], and the signal peptide hydrophobicity was determined according to Kyte&Doolittle using ProtScale [44, 45]. The Pearson correlation coefficient [46] was determined with the “Pearson function” within Microsoft Excel.

## Results and discussion

### Monitoring secretion stress response towards Cut11 secretion with the biosensor strain *B. subtilis* PAL5

The secretion stress biosensor strain *B. subtilis* PAL5 is based on the CssRS two-component system and was constructed by replacing the *htrA* gene with *mCherry* encoding the red-fluorescent protein to distinguish the biosensor signal from the green fluorescence signal of the split GFP assay.

**Fig. 1:**
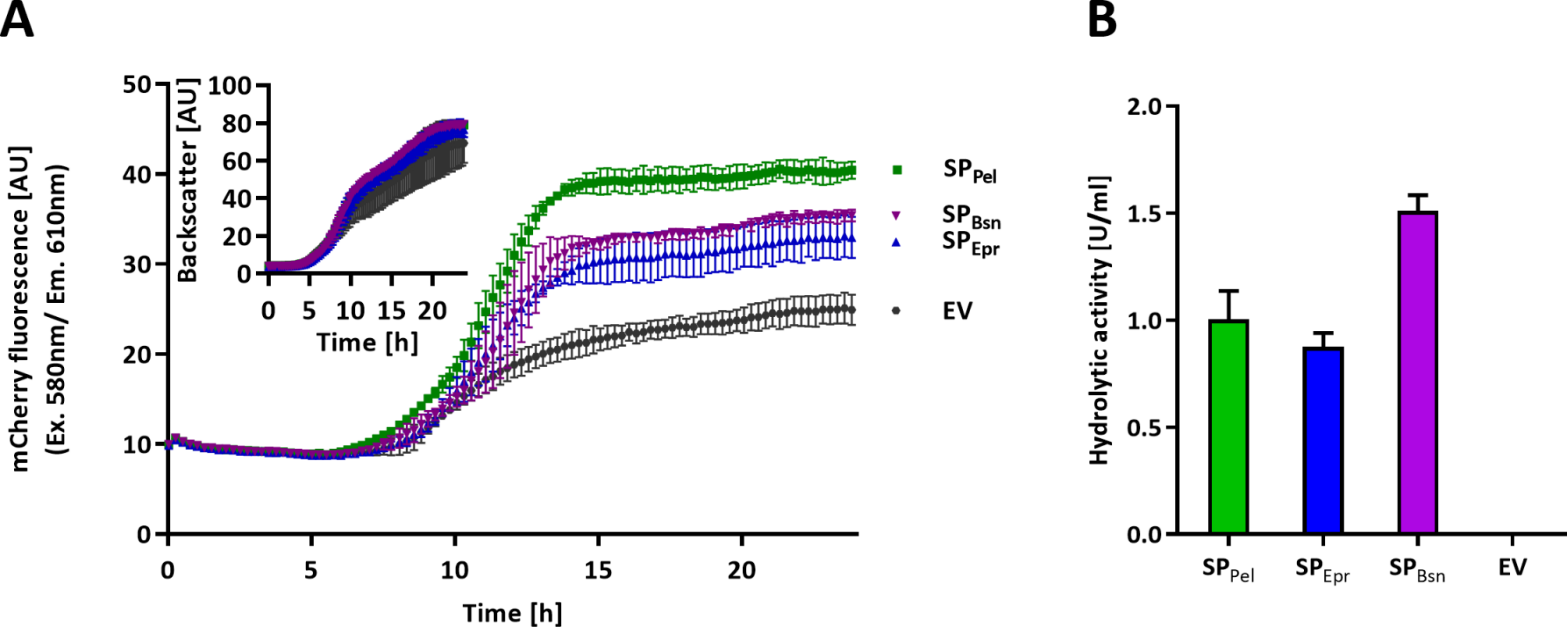
Stress response induced by secretion of cutinase fused to different signal peptides. **A** Growth and secretion stress were measured online in a Biolector microbioreactor. *B. subtilis* PAL5 harboring the expression vectors pBS-4nt-SP_Pel_-Cut11 (SP_Pel_), pBS-4nt-SP_Epr_-Cut11 (SP_Epr_) or pBS-4nt-SP_Bsn_-Cut11 (SP_Bsn_) for cutinase secretion or the empty vector (EV) was grown for 24 h and mCherry fluorescence (Ex. λ = 580 nm, Em. λ = 610 nm) as well as cell density (insert: light scattering at λ = 620 nm) were determined. Expression vectors harbor the constitutive P_HpaII_ promoter, a four nucleotide long ribosome binding site spacer between Shine-Dalgarno sequence and start codon and the GFP11 tag encoding sequence at the 3’-end of the *cut* gene. **B** Enzymatic activities in supernatants from BioLector grown cultures were determined with *p*NPP as substrate. Cultivation was carried out in biological triplicates and hydrolytic activity was measured in technical triplicates. Error bars indicate the standard deviations

The functionality of the biosensor strain was tested in a BioLector microbioreactor by time-resolved secretion stress analysis of *F. solani pisi* cutinase fused to three different *B. subtilis* signal peptides (see Additional file 1: Fig. S1), namely SP_Epr_ from the extracellular protease Epr [47], SP_Pel_ from the pectate lyase Pel [48] and SP_Bsn_ from the extracellular ribonuclease Bsn [49]. We previously observed that these signal peptide-cutinase fusions resulted in different amounts of extracellular cutinase in the order of SP_Pel_ > SP_Bsn_ > SP_Epr_ [29]. All variants were constitutively expressed in the biosensor strain *B. subtilis* PAL5. For online monitoring of secretion stress, transformants were cultivated in the BioLector microbioreactor for 24 h at 30 °C, while scattered light intensity and mCherry fluorescence were continuously measured (Fig. 1A). All cutinase producing cultures showed similar growth behavior (Fig. 1A, inset), which allowed a direct comparison of mCherry fluorescence intensities of the different strains. In all cases, secretion stress increased from the beginning of the exponential growth phase (approx. 6 h after inoculation) to around 12 h after inoculation (Fig. 1A). *B. subtilis* PAL5 had a relatively high basal stress level, as indicated by the fluorescence intensity of the empty vector harboring strain, presumably due to misfolding of naturally secreted proteins of *B. subtilis* as previously described [25].

The mCherry fluorescence for the variants with SP_Epr_ and SP_Bsn_ was similar throughout the cultivation period. For the SP_Pel_ variant, secretion stress increased slightly earlier and more strongly, resulting in about 30% higher mCherry fluorescence compared to the other cutinase-secreting strains. For analyzing the correlation between secretion stress and amount of active target protein after secretion, cutinase activities were measured with *p*NPP in the cell-free supernatants after cultivation (Fig. 1B). The SP_Epr_ variant exhibited the lowest stress level and also showed the lowest extracellular cutinase activity. Secretion of the variant SP_Bsn_ showed a similar stress response but yielded about 50% higher enzymatic activity. Variant SP_Pel_ clearly showed the highest stress level compared to the other signal peptide constructs, but enzymatic activity was only slightly higher than that of variant SP_Epr_ (1.00 ± 0.13 U/ml compared to 0.88 ± 0.06 U/ml). In contrast to our previous study [29], variant SP_Bsn_ showed a higher cutinase activity than variant SP_Pel_ presumably caused by different medium composition, here 8-fold more tryptone (8% (w/v) tryptone vs. 1% (w/v) tryptone) and different cultivation conditions (growth for 24 h at 30 °C vs. growth for 6 h at 37° C). Both factors strongly influence the protein yield as shown before for cutinase secretion by *C. glutamicum* mediated by different signal peptides [50].

Different signal peptides resulted in different yields of secreted enzyme; however, a correlation between the enzymatic activity and secretion stress was not observed, as it was already reported for *C. glutamicum* [24]. The reasons for this discrepancy can be manifold. SP_Pel_, for example, is a much shorter and less hydrophobic signal peptide than SP_Bsn_ and SP_Epr_ (see Additional file 1: Fig.S1). A similar signal peptide constellation has already been analyzed with the signal peptides of α-amylase, levansucrase, and levanase from *B. subtilis*. This comparison revealed that the signal peptide of levansucrase, which was the shortest and least hydrophobic signal peptide among the examined ones, is cleaved more slowly from the mature protein [51]. Furthermore, signal peptides also influence the translation initiation [29]. The tenfold higher translation initiation rate calculated for SP_Pel_ in comparison to SP_Bsn_ and SP_Epr_ could result in overloading of the chaperone PrsA, which has already been observed for the overexpression of other secretory proteins [52].

These results indicate that stress associated with Sec-dependent protein secretion can be easily monitored with the novel mCherry-based sensor strain *B. subtility* PAL5, thereby providing important information on possible bottlenecks of biotechnological production of secretory proteins in *B. subtilis*. On the other hand, the relation between stress response and amount of secreted enzyme or extracellular enzymatic activity remains to be elucidated in more detail.

### Online detection of secreted Cut11 during cultivation using the in vivo split GFP assay

The activity-independent split GFP assay provides information about the amount of cytoplasmic and secreted GFP11-tagged proteins [18, 19]. For the previously published in vitro split GFP assay, the cell-free culture supernatant that contains the GFP11 tagged target protein must be separated from cells after cultivation and subsequently mixed with externally produced GFP1-10 in solution. Before fluorescence measurements, this mixture has to be incubated for at least 16 h under agitation for reconstitution of fluorescent holo-GFP formed by GFP1-10 and the GFP11 tag [18]. In order to allow a fast detection suitable for online monitoring of target protein secretion, we tested the addition of GFP1-10 to the culture broth during cultivation thereby enabling in vivo holo-GFP reconstitution. To evaluate if the split GFP assay can also be applied in vivo for the online detection of protein secretion, *B. subtilis* PAL5 harboring one of the previously used expression plasmids was cultivated in a BioLector microbioreactor in enriched LB medium at 30 °C for 24 h. GFP1-10 solution was added directly to the culture broth at a final concentration of 3% (v/v) after 16 h of cultivation. Backscatter and GFP fluorescence were subsequently measured every 15 min for a time period of 24 h.

**Fig. 2:**
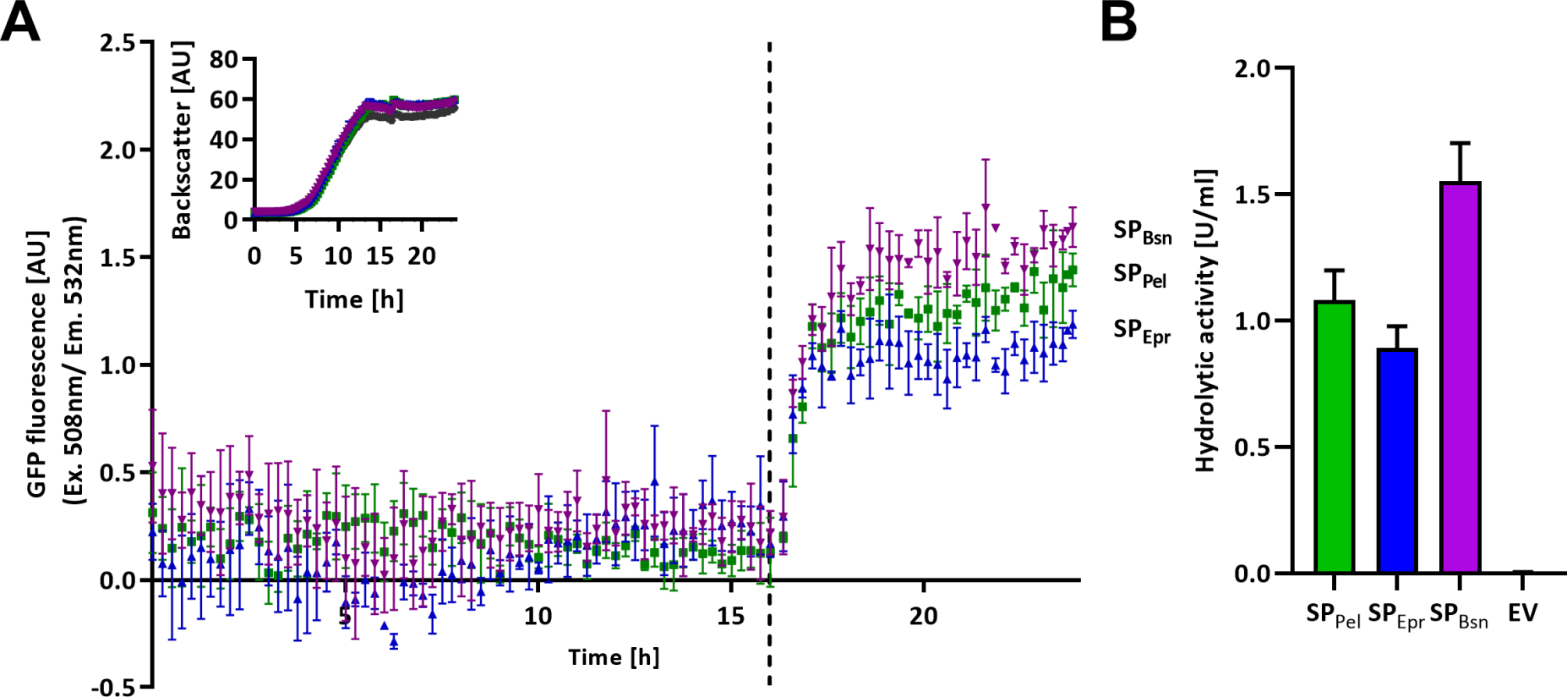
Online detection of Cut11 secretion using the in vivo split GFP assay. **A** Cell growth and online detection of secreted Cut11 were determined in a BioLector microbioreactor system. Cell density (light scattering at λ = 620 nm) and split GFP fluorescence (Ex. λ = 508 nm, Em. λ = 532 nm) were measured of *B. subtilis* PAL5 harboring one of the expression vectors pBS-4nt-SP_Pel_-Cut11 (SP_Pel_), pBS-4nt-SP_Epr_-Cut11 (SP_Epr_) or pBS-4nt-SP_Bsn_-Cut11 (SP_Bsn_) or the respective empty vector (pBSMul1, EV). All expression vectors harbor a four nucleotide spacer between Shine-Dalgarno sequence and *cut11* start codon. The fluorescence values of strains carrying the empty vector were subtracted from the fluorescence values of cutinase-expressing strains. A solution of GFP1-10 was directly added to the culture broth to a final concentration of 3% (v/v) after 16 h of cultivation (indicated by dotted line). **B** Enzymatic activities of supernatants of cultures grown for 24 h in a BioLector system. All cultivations were performed in biological triplicates and activity measurements were additionally performed in technical triplicates. Error bars indicate the standard deviations

We directly compared the fluorescence values determined for different strains producing SP_Pel_-, SP_Epr_ or SP_Bsn_-Cut11, as all cultures showed similar growth behavior (Fig. 2A, inset). The fluorescence values obtained from strains carrying the corresponding empty vector were subtracted from those of cutinase secreting strains (Fig. 2A). As expected, the target protein producing strains showed almost no fluorescence signal until the GFP1-10 solution was added. Then, GFP fluorescence rapidly increased for about an hour, caused by assembly of holo-GFP from GFP1-10 and secreted cutinase carrying the GFP11 tag. A good correlation was observed between split GFP fluorescence and the corresponding enzymatic activity determined with the substrate *p*NPP (Fig. 2B). The results therefore clearly demonstrate the applicability of the in vivo split GFP assay as an easy-to-perform and time-saving in vivo assay suitable for online detection of secreted enzymes.

### Analyzing cutinase secretion by simultaneous use of the split GFP assay and the secretion stress biosensor

**Fig. 3:**
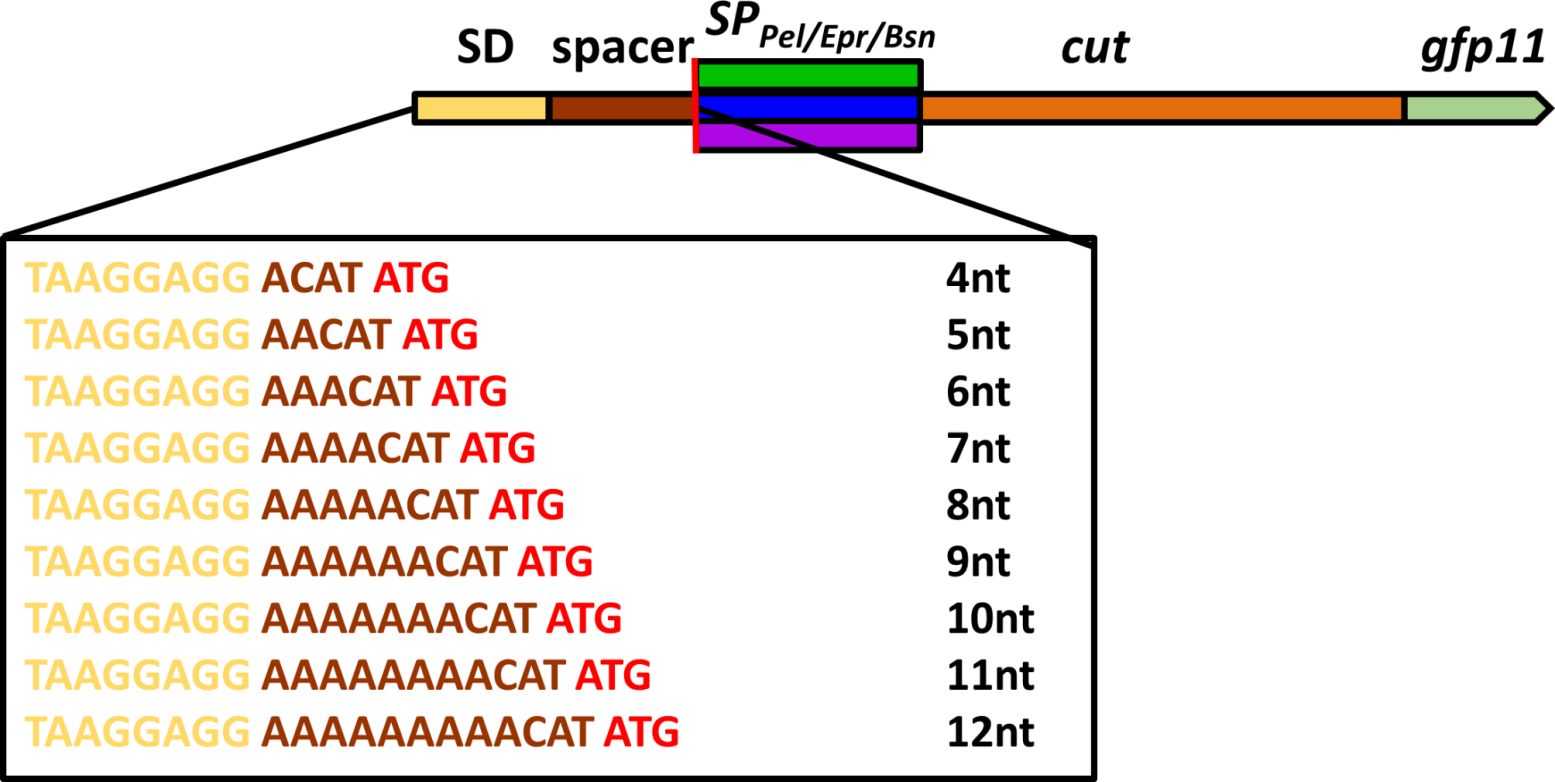
Schematic illustration of the cutinase expression plasmids. The expression plasmids harbor a Shine Dalgarno sequence (SD, yellow), a spacer region (brown), the start codon of the target gene (red line), the respective signal sequence (SP_Pel_ [dark green], SP_Epr_ [blue] or SP_Bsn_ [violet]), cut (orange) and the *gfp11 tag* (light green). The nucleotide sequences covered by the Shine Dalgarno (SD) sequence and the spacer region with lengths varying from 4 to 12 nucleotides (nt) by insertion of adenosines are shown

**Fig. 4:**
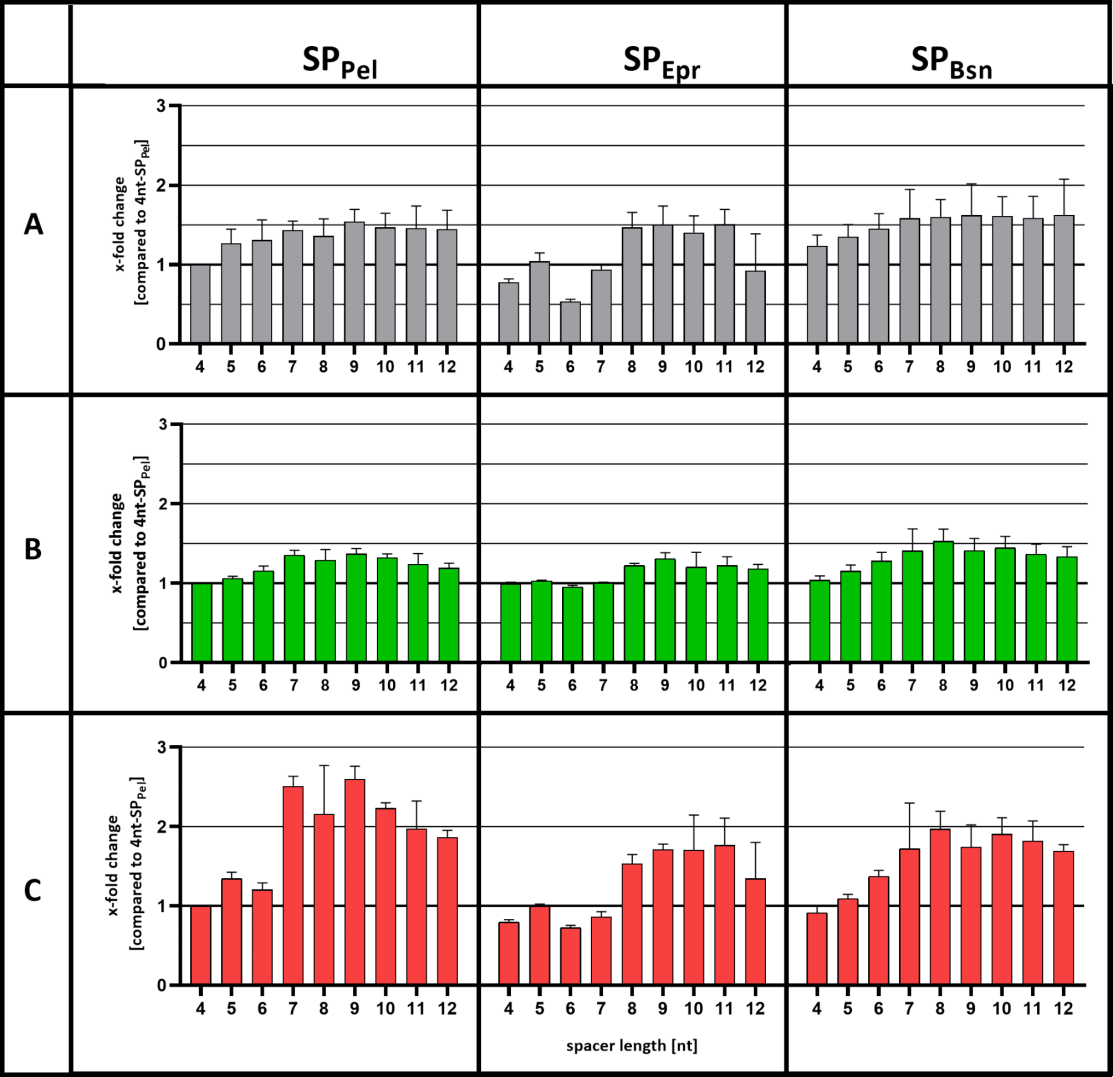
Analysis of cutinase secretion by B. subtilis with Cut11 carrying three different signal peptides and varying the length of the ribosome binding site spacers. *B. subtilis* PAL5 strains harboring expression plasmids pBS-Xnt-SP_Pel_-Cut11 (SP_Pel_), pBS-Xnt-SP_Epr_-Cut11 (SP_Epr_) or pBS-Xnt-SP_Bsn_-Cut11 (SP_Bsn_) were cultivated in a BioLector microbioreactor system at 30 °C and 1100 rpm for 24 h. The plasmid series differ in the length of the ribosome binding site spacer (4–12 nucleotides, indicated as Xnt in the plasmid name) between the Shine-Dalgarno sequence and the *cut11* start codon resulting in a gradual change of translation efficiency as described by Volkenborn et al., 2020. **A** Relative enzymatic activities (grey bars) were measured with the substrate *p*NPP. **B** Relative amounts of secreted Cut11 (green bars) determined by the in vivo split GFP assay, where a solution of 3% (v/v) GFP1-10 was directly added into the culture broth after 16 h of cultivation. **C** The change of secretion stress was determined as relative fluorescence of mCherry (red bars). All cutinase activities and fluorescence values of split GFP and mCherry were measured after 24 h of cultivation, normalized to the backscatter and compared to the 4nt-SPPel variant. Cultivations were performed in biological triplicates and activity measurements were additionally performed in technical triplicates. Error bars indicate the standard deviations

As a next step, we evaluated the simultaneous use of the mCherry-based secretion stress biosensor strain *B. subtilis* PAL5 and the in vivo split GFP assay to investigate whether the amount of secreted Cut11 (determined by the split GFP and activity assay) directly correlates with the corresponding signal of the secretion stress biosensor. To this end, the production of SP_Pel_-, SP_Epr_- and SP_Bsn_-Cut11 was gradually increased by varying the spacer length (four to twelve nucleotides) between the Shine-Dalgarno sequence and the Cut11 start codon binding site as described previously [29]; the constructs are shown in Fig. 3. The cutinase secreting strains were cultivated at 30 °C and 1100 rpm for 24 h, the fluorescence of holo-GFP and mCherry were measured directly in the culture broth after cultivation, while the enzymatic activities were measured in the cell-free supernatant. All values were normalized to the SP_Pel_ variant with a four nucleotide long spacer (4nt-SP_Pel_; Fig. 4).

As expected, Cut11 secretion for each signal peptide was maximally increased when the spacer length was extended from 4 to 8 or 9 nt, as observed previously [29]. A good correlation was found between fluorescence data from the in vivo split GFP assay and the enzymatic activities (Fig. 4A and B; r = 0.85; R^2^ = 0.73). However, the sensitivity of the in vivo split GFP assay decreased with inferior performing variants, as shown by similar split GFP fluorescence, but different activities, e.g., for the variants carrying the signal peptide SP_Epr_ and 4 to 6 nt spacers (Fig. 4B-SP_Epr_; see Additonal file 1: Tab. S1). Notably, the enzymatic activities and the stress responses of *B. subtilis* strains secreting cutinase fused to different signal peptides and with varying spacer lengths showed a good correlation (Fig. 4A and C; see Additional file 1: Tab. S2). The correlation between secretion stress response and enzymatic activities was similar for cutinases which secretion was facilitated by the signal peptides SP_Epr_ and SP_Bsn_ (r = 0.92 and r = 0.96). For SP_Pel_ the correlation between biosensor response and enzymatic activity was slightly worse (r = 0.84). Here, it was noted that for spacer lengths ≥ 7 nt the increase of secretion stress response was higher than the increase in cutinase amount and enzymatic activity. The level of secretion stress determined for the constructs carrying different spacer lengths coincided with the corresponding calculated translation initiation rates that we have reported previously (see [29] for details). The disproportionally high increase in secretion stress may indicate improper protein folding at a significant rate, thereby reducing efficient protein secretion. Excessive protein secretion can result in overloading the secretion machinery, but also protein folding components, as shown for the extracellular chaperone PrsA [52]. Additional experiments are required to prove if this is the case here.

In conclusion, we have successfully combined the split GFP assay with the secretion stress biosensor thereby providing more detailed insights into the process of recombinant protein secretion. Increased stress can pinpoint a bottle neck in secretion; therefore, it is desirable for secretion optimization to select a SP that confers low accompanying secretion stress.

### Screening of a signal peptide library using the in vivo split GFP assay and the secretion stress biosensor strain *B. subtilis* PAL5

**Fig. 5:**
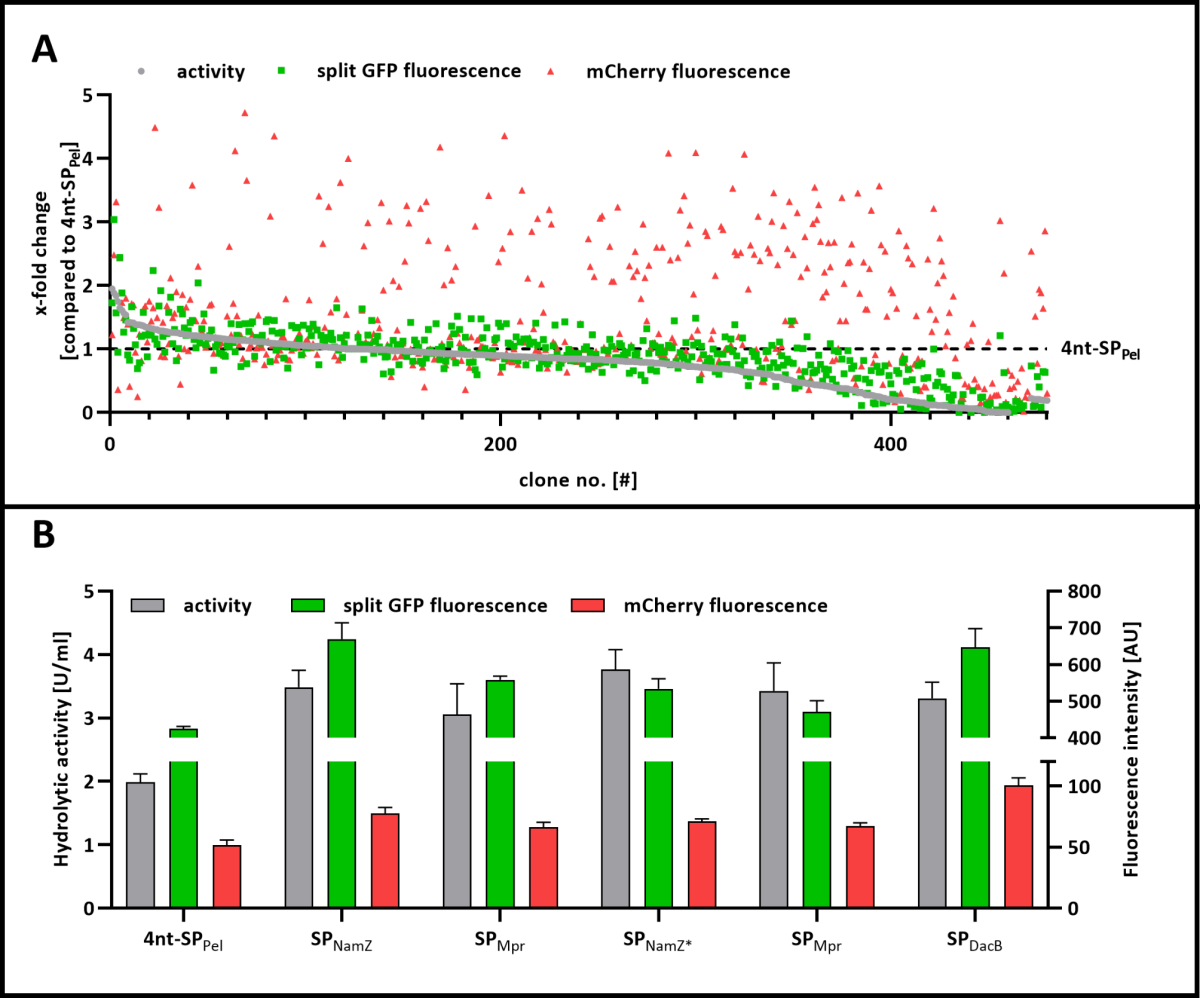
Screening of a SP-Cut11 library. **A** The *cut11* gene was fused to *B. subtilis* signal peptide library. The secretion biosensor *B. subtilis* PAL5 was transformed with the SP library and 480 clones were analyzed in vivo regarding the enzymatic activity using the substrate *p*NPP (grey, activity), the protein amount as determined by split GFP fluorescence (green, split GFP fluorescence) and the corresponding secretion stress as indicated by mCherry fluorescence (red, mCherry fluorescence) The construct 4nt-SP_Pel_-cutinase was taken as benchmark (dotted line) **B** Selected variants were transferred to *B. subtilis* PAL5 and the resulting strains were cultivated, analyzed, and the corresponding signal peptides were identified by DNA sequencing

Finally, we analyzed, whether the combination of the detection systems described before is applicable for a screening campaign at 96 well-scale by using the cutinase as a model enzyme. To this end, the *cut11* gene was fused to all 173 -Sec-related signal peptides predicted for proteins secreted by *B. subtilis* [13]. After transformation of *B. subtilis* PAL5 with the SP-Cut11 library, 480 individual clones were analyzed with respect to the amount and activity of the secreted cutinase and the accompanying secretion stress, as determined by measuring the mCherry fluorescence. The data were normalized to those obtained with *B. subtilis* PAL5 expressing the fusion 4nt-SP_Pel_^−^cutinase defined as benchmark (Fig. 5A).

Screening of the signal peptide library again revealed a correlation between results obtained with the split GFP assay and enzymatic activity (r = 0.77; R^2^ = 0.61), although with reduced sensitivity for low-secreting variants. Again, no correlation was observed between the secretion stress response and the amount/activity of the secreted cutinase. Screening campaigns with single biological samples are however subject to large statistical variations. Therefore, we selected five clones with the highest level of cutinase secretion based on the split GFP measurement that were subsequently recultivated as biological triplicates and then reassessed for cutinase secretion and associated stress (Fig. 5B).

All variants showed similar enzymatic activities and split GFP fluorescence intensities. Plasmid DNA sequencing of the respective five parental clones identified the signal peptides SP_NamZ_ (and SP_NamZ*_), SP_Mpr_ (two clones) and SP_DacB_, responsible for the high-level cutinase secretion. The split GFP fluorescence of variant SP_NamZ*_-cutinase was slightly reduced compared to SP_NamZ_-cutinase. It is interesting to note that SP_NamZ*_-cutinase harbors the point mutation A252S in the GFP11 tag, which may affect holo-GFP reconstitution. Among the variants, SP_DacB_-cutinase and SP_NamZ_-cutinase were the best secreted enzymes and they were secreted at a similar level. Considering the cellular stress associated with secretion, a substantially higher mCherry fluorescence was detected for SP_DacB_ (100.48 ± 6.26 AU vs. 77.71 ± 4.76 AU, respectively) indicating a more pronounced stress response. Apparently secretory production of SP_NamZ_-cutinase poses less stress on the cells, suggesting more efficient secretion. Thus, for further optimization of secretion, for instance by improving the signal peptide *via* (random) mutagenesis approaches SP_NamZ_ would be the preferred choice. However, in case variant SP_DacB_-cutinase would be chosen for further optimization it is desirable to also alleviate increased secretion stress e.g. by optimization of the secretory protein folding for instance by enrichment of cations in the peptidoglycan layer or by overexpression of the molecular chaperone PrsA [53–55].

In conclusion, we showed here for the secretion of cutinase by *B. subtilis* that the secretion stress response only partially reflects secretion levels. Protein misfolding competes with effective secretion leading to high-level stress and disproportionally low protein secretion. Nevertheless, the stress response can pinpoint secretion bottlenecks and thus can aid in selection of a more suitable signal peptide for further improvement of secretion of a target protein.

## Conclusions

In this study, we have combined the mCherry-based secretion stress biosensor strain *B. subtilis* PAL5 with the in vivo split GFP assay allowing for parallel monitoring of the cellular stress response during protein secretion and the activity-independent quantification of a secreted target protein. The in vivo split GFP assay consistently revealed a good correlation with extracellular enzymatic activity, although a slightly reduced sensitivity was observed for variants exhibiting low secretion levels. Interestingly, we did not observe a direct correlation between secretion stress and the amount of secreted protein fused to different signal peptides. The signal peptide (and its corresponding RNA regions) may affect diverse steps on the way to the mature secreted enzyme, e.g., mRNA stability, translation initiation, interaction with the signal recognition particle, signal peptide processing and folding upon/after translocation. Upon modulation of the translation initiation rate by changing the length of the ribosomal binding site spacer [29], we indeed observed a correlation between the amount and activity of secreted target protein and secretion stress. Our results suggest that the secretion biosensor strain *B. subtilis* PAL5 can be used in combination with fluorescence activated cell sorting (FACS) for the identification of cells, which secrete a protein of interest at high level as previously demonstrated for the secretion biosensor strain *C. glutamicum* K9 using a signal peptide mutant library [15]. As a future approach it appears conceivable to combine the here described methods with the previously described iSplit GFP assay [19] for the in vivo detection of intracellular target proteins. Such a combination would allow to monitor in more detail the entire secretion process and thus enable researchers to easily identify bottlenecks for secretion optimization.

### Electronic supplementary material

Below is the link to the electronic supplementary material.

Supplementary Material 1

## Data Availability

The datasets supporting the conclusions of this article are included within the article and its additional file.
