# Supplementary material for: Analysis of protein secretion in Bacillus subtilis by combining a secretion stress biosensor strain with an in vivo split GFP assay
Source: Microb Cell Fact. 2023 Oct 7;22:203. doi: 10.1186/s12934-023-02199-8 (PMC10559633; doi:10.1186/s12934-023-02199-8)
Supplement: Supplementary file 1 — Supplementary Material 1 [file 12934_2023_2199_MOESM1_ESM.docx]

# Supplementary File 1

**Analysis of protein secretion in *Bacillus subtilis* by combining a secretion stress biosensor strain with an *in vivo* split GFP assay**

Patrick Lenz^1,5^, Patrick J. Bakkes^2,5^, Carolin Müller^2,3^, Marzena Malek^1^, Roland Freudl^2^,

Marco Oldiges^2,3^, Thomas Drepper^1^, Karl-Erich Jaeger^1,2*^, Andreas Knapp^1,4*^

^1^Institute of Molecular Enzyme Technology, Heinrich Heine University Düsseldorf, Forschungszentrum Jülich, 52428 Jülich, Germany

^2^Institute of Bio- and Geoscience, IBG-1: Biotechnology: Forschungszentrum Jülich, 52425 Jülich, Germany

^3^Institute of Biotechnology, RWTH Aachen University, 52074 Aachen, Germany

^4^present address: Castrol Germany GmbH, 41179, Mönchengladbach, Germany

^5^present address: Institute of Biotechnology, RWTH Aachen University, 52074 Aachen, Germany

#

Fig. S1. Signal peptide analysis. **A** Schematic model of the domain structure of signal peptides SP_Pel_, SP_Epr_ and SP_Bsn_. The amino acid sequence, its length and subdivision into N-(blue), H- (yellow) and C-region (gray) predicted by SignalP 6.0 (Teufel *et al.*, 2021) are shown. **B** Hydrophobicity plots predicted by ProtScale for the selected signal peptides using the hydropathy scale according to Kyte and Doolittle (Gasteiger *et al.*, 2005).

Tab. S1: Relative split GFP fluorescence and relative enzymatic activity taken from data in Fig. 3 to illustrate the reduced sensitivity of the *in vivo* split GFP assay.

|  | **4nt-SP_Epr_** | **5nt-SP_Epr_** | **6nt-SP_Epr_** |
| --- | --- | --- | --- |
| **Holo-GFP fluorescence [compared to 4nt-SP_Pel_]** | 1.00 ± 0.02 | 1.03 ± 0.01 | 0.95 ± 0.02 |
| **Activity [compared to 4nt-SP_Pel_]** | 0.78 ± 0.04 | 1.04 ± 0.10 | 0.54 ± 0.03 |

Tab. S2: Calculated correlations and associated coefficients of determination between hydrolytic activities and secretion stress response with varying ribosome binding site spacers.

|  | **Xnt-SP_Pel_** | **Xnt-SP_Epr_** | **Xnt-SP_Bsn_** |
| --- | --- | --- | --- |
| **Correlation [r]** | 0.84 | 0.92 | 0.96 |
| **Coefficient of determination [R^2^]** | 0.71 | 0.85 | 0.92 |

# References

Gasteiger, E., Hoogland, C., Gattiker, A., Wilkins, M. R., Appel, R. D. & Bairoch, A. 2005. Protein identification and analysis tools on the ExPASy server. *The proteomics protocols handbook***,** 571-607.

Teufel, F., Armenteros, J. J. A., Johansen, A. R., Gíslason, M. H., Pihl, S. I., Tsirigos, K. D., Winther, O., Brunak, S., von Heijne, G. & Nielsen, H. 2021. SignalP 6.0 achieves signal peptide prediction across all types using protein language models. *bioRxiv***,** 2021.06.09.447770.
